# Spiers Memorial Lecture: Shielding the active site: a streptavidin superoxide-dismutase chimera as a host protein for asymmetric transfer hydrogenation†

**DOI:** 10.1039/d3fd00034f

**Published:** 2023-03-16

**Authors:** Nico V. Igareta, Ryo Tachibana, Daniel C. Spiess, Ryan L. Peterson, Thomas R. Ward

**Affiliations:** a Department of Chemistry, University of Basel Mattenstrasse 24a, BPR 1096 Basel CH-4058 Switzerland thomas.ward@unibas.ch; b National Center of Competence in Research (NCCR) “Molecular Systems Engineering” 4058 Basel Switzerland rlp139@txstate.edu

## Abstract

By anchoring a metal cofactor within a host protein, so-called artificial metalloenzymes can be generated. Such hybrid catalysts combine the versatility of transition metals in catalyzing new-to-nature reactions with the power of genetic-engineering to evolve proteins. With the aim of gaining better control over second coordination-sphere interactions between a streptavidin host-protein (Sav) and a biotinylated cofactor, we engineered a hydrophobic dimerization domain, borrowed from superoxide dismutase C (SOD), on Sav’s biotin-binding vestibule. The influence of the SOD dimerization domain (DD) on the performance of an asymmetric transfer hydrogenase (ATHase) resulting from anchoring a biotinylated Cp*Ir-cofactor – [Cp*Ir(biot-*p*-L)Cl] (1-Cl) – within Sav-SOD is reported herein. We show that, depending on the nature of the residue at position Sav S112, the introduction of the SOD DD on the biotin-binding vestibule leads to an inversion of configuration of the reduction product, as well as a fivefold increase in catalytic efficiency. The findings are rationalized by QM/MM calculations, combined with X-ray crystallography.

## Abstract 2

We have evaluated chimeric streptavidin superoxide dismutase C as a scaffold for an asymmetric transfer hydrogenase, incorporating [Cp*Ir(biot-*p*-L)Cl] as a cofactor.

## Graphical abstract

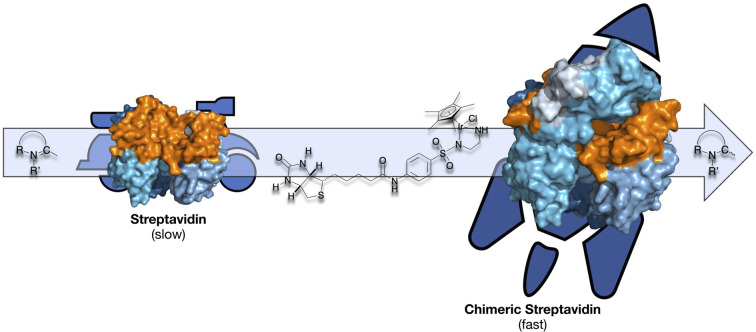

## Introduction

**Scheme 1:**
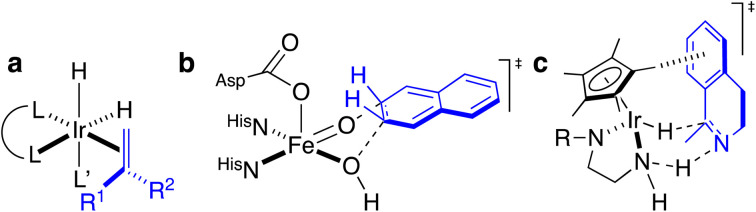
Homogeneous and enzymatic catalysts often rely on different enantiodiscriminating mechanisms. (a) Ir-catalyzed hydrogenation as prototypical homogeneous catalysis: such systems often rely on the coordination of the prochiral substrate (blue) to the metal prior to the enantiodetermining step. (b) Thanks to a well-structured second coordination sphere around the metal cofactor, a substrate (blue) need not be coordinated to the metal to undergo enantiospecific derivatization. (c) Postulated transition state for the three-legged piano-stool catalyzed asymmetric transfer hydrogenation. As can be appreciated, the prochiral imine (blue) does not bind to the d^6^-metal, highlighting the importance of second coordination sphere interactions in the enantiodiscriminating step.

Artificial metalloenzymes (ArMs) consist of a catalytically-competent cofactor anchored within a protein host.^1,2^ These systems uniquely combine the synthetic tunability of homogenous catalysis with the evolutionary malleability of nature’s fundamental protein building blocks to yield new chemistries. One of the first ArMs was reported by Wilson and Whitesides in 1978. They demonstrated that anchoring an achiral biotinylated rhodium cofactor in avidin affords a hybrid catalyst for the asymmetric hydrogenation of prochiral alkenes.^3^ Since that seminal report, advancements in the fields of homogenous catalysis and molecular biology have resulted in the development of a large variety of ArMs featuring different cofactors and diverse protein scaffolds, including carbonic anhydrase,^4^ hemoproteins,^5–7^ prolyl oligopeptidase,^8^ nitrobindin,^9^ human serum albumin,^10^ (strept)avidin, *etc.*^1,3,11^ Such ArMs combine attractive features from both organometallic catalysts and enzymes. For example, thanks to the availability of the entire periodic table to select a suitable metal, organometallic catalysts enable access to new-to-nature reactions. However, the second coordination sphere around a small molecule catalyst is challenging to tailor at will. Accordingly, many highly selective metal-catalyzed reactions rely on the binding and activation of substrates at the metal (Scheme 1a). In contrast, as metal cofactors are often deeply embedded within a protein scaffold, second coordination sphere interactions between a substrate and the host protein can be tailored by genetic means. This asset is reflected in the mechanism of many (metallo)enzymes, whereby the substrate need not bind to the cofactor prior to functionalization (Scheme 1b).

**Fig. 1:**
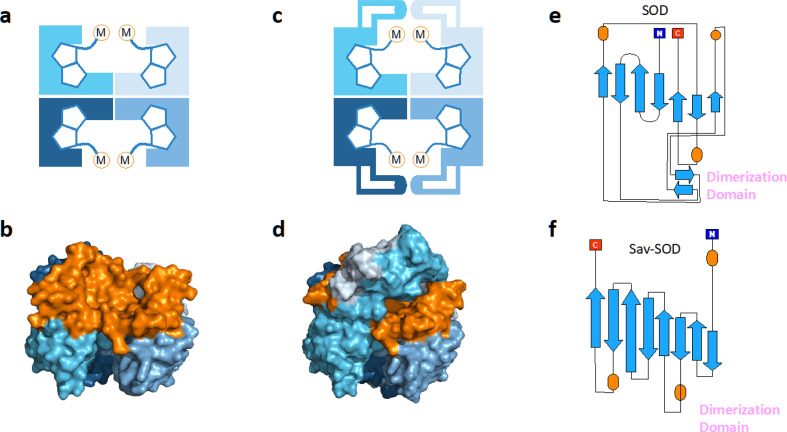
(a) Schematic representation of tetrameric streptavidin (Sav) and (b) surface representation of Sav (pdb 3pk2) centered on one of the two biotin binding vestibules; each monomer is represented by a different tone of blue and the surface of the vestibule is highlighted in orange. (c) Schematic representation of Sav-SOD, illustrating the potential shielding of the cofactor and (d) surface representation of Sav-SOD centered on the biotin-binding vestibule, highlighting the shielding of the vestibule (orange surface) by the DD (turquoise and grey surface). Origami representations of the monomer of (e) SOD including the DD between β-sheets 8 and 9 (highlighted with pink text) and (f) chimeric Sav-SOD including the DD inserted between β-sheets 3 and 4. Alpha helices are represented as orange ovals.^22^

Over the last two decades, the Ward group has extensively capitalized on streptavidin (Sav)–biotin technology to create a wide range of ArMs exhibiting unique biochemistry.^11–19^ However, due to the inherent topology of Sav, the metal cofactors remain relatively solvent-exposed and accessible (Fig. 1a and b), whereas in many natural metalloenzymes, the cofactors (*e.g.*, metal ions, heme, pterins, *etc.*) are often deeply buried within their host protein. A significant advantage of this feature is that the secondary coordination sphere interactions can be exploited to alter cofactor reactivity and/or enhance substrate modification with very high selectivity (Scheme 1b).

Our group has explored numerous strategies for enclosing the streptavidin biotin-binding vestibule in order to furnish corresponding ArMs with a more defined substrate-binding pocket. These strategies have included the insertion of (GGS)_*n*_ loop extensions, the use of chimeric-Sav genes containing small synthetic and natural peptide domains ranging from 24 to 60 residues in size, and the encapsulation of Sav within proteins or nanoparticle host scaffolds.^20,21^ However, these approaches are not amenable to high-throughput methodologies because of limited protein production levels or necessary time-consuming refolding and/or extensive processing workflows. To circumvent the challenges faced with previous Sav designs, we recently introduced a chimeric streptavidin variant (Fig. 1c and d) as a scaffold for an artificial hydroaminase (HAMase) based on dual-gold activation of alkynes.^22^ In that study, we introduced the dimerization domain (DD) of the superoxide dismutase C (SOD) from *M. tuberculosis* (pdb 1pzs)^23^ (Fig. 1e) into the 3,4-loop of streptavidin (Fig. f). By directed evolution, the second coordination sphere around the abiotic cofactor was further optimized to control the regioselectivity of the hydroamination reaction, leading to two mutants for either a single gold π-activation or a dual gold σ,π-activation of the alkyne substrate.

Encouraged by these results, we sought to utilize the Sav-SOD chimera as a protein for the asymmetric transfer hydrogenation catalyzed by a biotinylated d^6^-piano stool complex [Cp*Ir(biot-*p*-L)Cl] (1-Cl) as a cofactor. As highlighted in Scheme 1c, the prochiral substrate does not bind to the piano stool catalyst prior to the delivery of the hydride. We thus speculated that the presence of a well-structured and shielded second coordination sphere provided by Sav-SOD should significantly affect the catalytic performance of the resulting asymmetric transfer hydrogenase (ATHase hereafter). Herein, we present the results of our investigation for the reduction of prochiral cyclic imines.

## Results and discussion

### Biochemical properties of the Sav-SOD chimera

Initial protein-expression experiments revealed that the chimera with the DD inserted into the 3,4-loop yielded a soluble protein that displayed biotin-binding properties. Analysis of the chimeric Sav-SOD by native mass spectrometry confirmed expression fidelity.^22^ Analysis of the binding of 1-Cl within Sav and Sav-SOD by isothermal titration calorimetry (ITC) revealed very similar binding properties: 8.7 nM (*K*_D_Sav_) and 7.5 nM (*K*_D_Sav-SOD_) (Fig. S1†). With the chimeric Sav in hand, we sought to investigate the versatility of the Sav-SOD scaffold for the development of an artificial transfer hydrogenase (ATHase) featuring 1-Cl as a cofactor.

### Impact of the SOD domain on ATHase activity

**Fig. 2:**
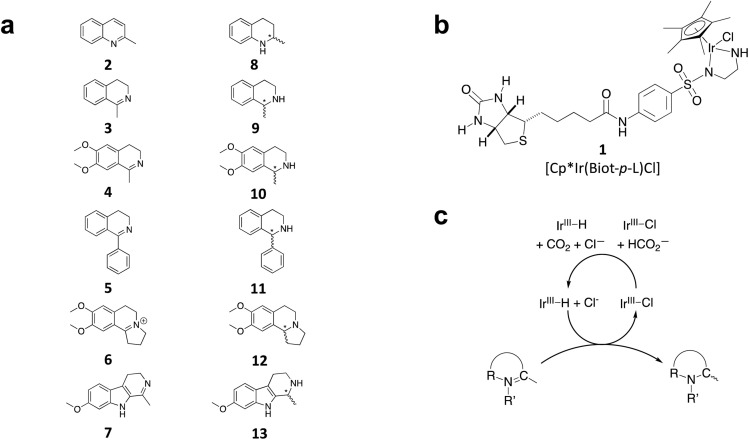
(a) Structure of the cyclic imine substrates (2–7) used for the ATHase studies with their corresponding enantioenriched products (8–13). (b) Structure of the cofactor [Cp*Ir(biot-p-L)Cl] (1-Cl) used in this study. (c) Schematic representation of the transfer hydrogenation mechanism.

We selected six structurally-related bicyclic prochiral imines (2–7) that afford the corresponding enantioenriched amines (8–13) upon asymmetric transfer hydrogenation (Fig. 2a).^13,24^ The ATHase based on cofactor 1-Cl anchored within Sav (1-Cl·Sav hereafter, Fig. 2b) has been widely-applied, in the presence of sodium formate as a hydride source.^13,24–28^ For this system, the stepwise reaction involves the formation of Ir^III^–H by hydride transfer from formate, followed by hydride transfer to the prochiral imine carbon, yielding the enantioenriched amine (Fig. 2c). The C–H bond-forming reaction has been proposed to occur *via* a non-concerted mechanism involving a CH⋯π interaction between the substrate and the Cp* ligand of the cofactor,^24^ and it has been shown that the Sav host can impact substrate turnover and enantioselectivity.^13,24–27,29–31^ Most notably, a single alanine substitution targeting residues S112 and K121, located within 5–10 Å of the biotin vestibule, has been shown to significantly impact ATHase activity.^13,25,32^ Thus, we initiated our Sav-SOD chimera studies with single alanine mutants targeting either the S112 or K121 position. For simplicity, the amino acid numbering for Sav-SOD was kept the same as for Sav, despite the presence of the 34 amino acids from SOD in the 3,4-loop (Fig. 1).

Mass spectrometry was used to confirm the ATHase assembly resulting from the incorporation of 1 within Sav-SOD S112A and Sav-SOD K121A, yielding 1-Cl·Sav-SOD S112A and 1-Cl·Sav-SOD K121A, respectively. We observed molecular ion peaks of ∼797 *m*/*z* units per equivalent of 1 bound to each tetrameric Sav-SOD host, corresponding to cofactor 1 –with no Cl^−^ bound – anchored within its respective biotin-binding host protein (Fig. S2†). Both Sav-SOD ATHases reduced the prochiral imine substrates in excellent yields in the presence of sodium formate as a sacrificial hydride donor, at 37 °C and pH 6.0–7.0. The results from the activity assay using the ATHases, as well as the free cofactor 1-Cl, are collected in Table 1. In all cases, the turnover number (TON) achieved with the Sav-SOD ATHases is comparable to or exceeds that achieved with the benchmark Sav-based ATHases.

**Table 1:** Selected results for ATHase variants in the presence of either Sav or Sav-SOD as host protein. The reactions were performed with 10 mM substrate at 37 °C for 48 h (see ESI† for details). The conversion is displayed with the enantioselectivity in parentheses. Positive % ee values correspond to the (*R*)-product and negative % ee values correspond to the (*S*)-product. For product 12, the absolute configuration was not determined

	Product	No protein	Sav WT	Sav S112A	Sav K121A	Sav-SOD S112A	Sav-SOD K121A
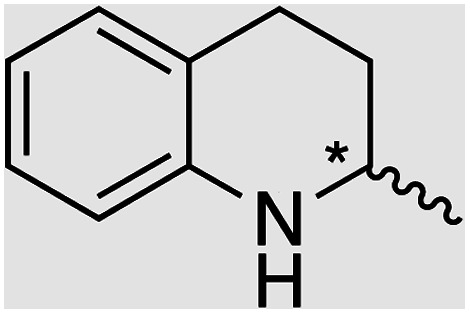	8	44 (−4)	49 (−71)	46 (−68)	48 (−46)	47 (−20)	70 (−77)
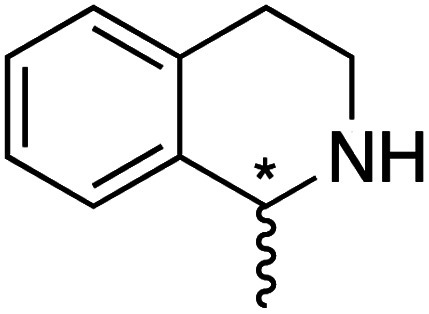	9	28 (−5)	63 (41)	74 (20)	93 (28)	84 (62)	100 (59)
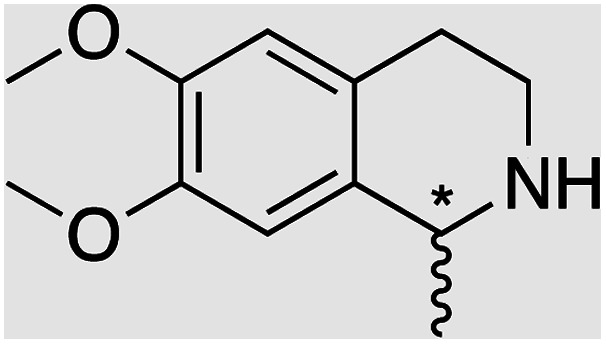	10	82 (−2)	62 (29)	84 (75)	96 (9)	100 (69)	100 (−5)
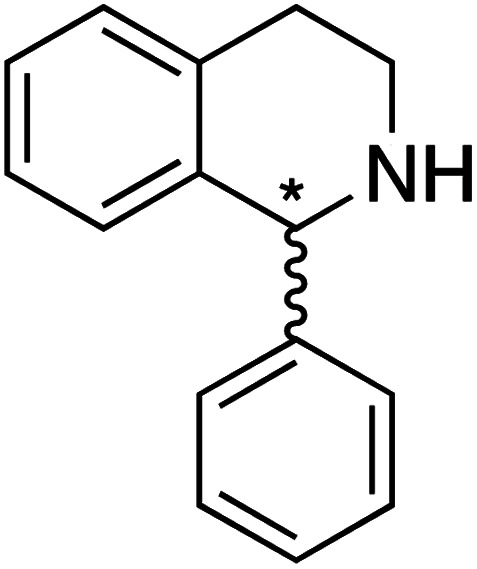	11	58 (−1)	80 (58)	78 (64)	40 (66)	77 (−27)	86 (58)
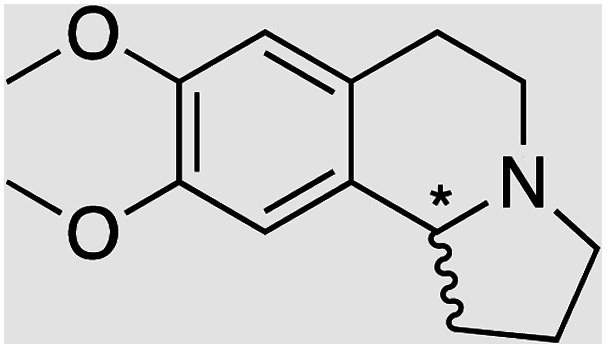	12	6 (4)	7 (−7)	7 (−5)	7 (2)	21 (−57)	9 (−36)
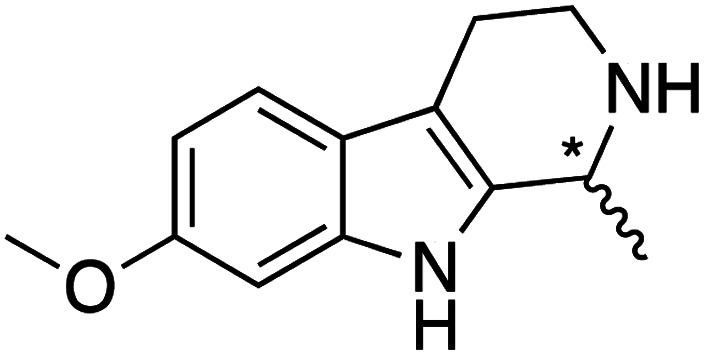	13	87 (−2)	87 (−25)	94 (32)	100 (−18)	100 (58)	100 (37)

For the structurally-related quinoline and dihydroisoquinoline substrates bearing a methyl substituent on the prochiral imine (2, 3, and 4), the preferred enantiomers for products (8, 9, and 10), formed with either Sav-SOD or the related Sav ATHases, were the same. In contrast, an inversion of the preferred enantiomer was observed for substrate 5, which possesses a bulky phenyl moiety at its prochiral imine carbon. In particular, 1-Cl·Sav S112A yielded primarily the (*R*)-11 product (64% ee) in 78% yield, while 1-Cl·Sav-SOD S112A afforded the (*S*)-11 product (27% ee) in 77% yield. Strikingly, 1-Cl·Sav K121A and 1-Cl·Sav-SOD K121A produce the same (*R*)-11 product with 66% and 58% ee, respectively. Importantly, the conversion after 48 hours achieved by 1-Cl·Sav-SOD K121A is more than double that achieved by 1-Cl·Sav K121A (86% *vs.* 40%).

From previous crystallographic analysis of 1-Cl·Sav S112A (pdb 3pk2), combined with quantum mechanics/molecular mechanics (QM/MM) studies, we suggested that the reduction of 4 proceeds through a non-concerted mechanism involving a CH⋯π interaction between the substrate and [Cp*Ir(biot-*p*-L)H] (1-H), with possible involvement of residue K121 in the protonation step.^24,33,34^ Based on the trends observed for substrates 2–7, we hypothesize that the DD of Sav-SOD renders the ATHase activity and selectivity more responsive to subtle modifications, thus highlighting the influence of the DD on the positioning of the prochiral substrate within the biotin-binding vestibule. The increased yields obtained with the Sav-SOD ATHases may result from the more hydrophobic catalytic site.^22^ This is in line with the previous observation that the activity of 1-Cl·Sav was positively affected by the mutation of the cationic residue at position 121 into a hydrophobic residue.^29^

Distinctions between the Sav and Sav-SOD ATHases are more apparent for the crispine A precursor (6) and harmaline (7). Cationic substrate 6 is structurally related to 4 but contains a bicyclic iminium moiety. Most notably, 1-Cl·Sav-SOD S112A produces 12 with 57% ee, compared to the modest 5% ee observed when using 1-Cl·Sav S112A. Furthermore, the chimeric streptavidin converted 3 times more substrate than the cofactor alone (6% conv. *vs.* 21% conv.). With the ring-expanded harmaline (7), we observed a moderate level of enantioselectivity for the dihydroharmaline product 13 with 1-Cl·Sav S112A ((*R*)-13 with 32% ee) and 1-Cl·Sav K121A ((*S*)-13 with 18% ee). However, both Sav-SOD S112A and Sav-SOD K121A-based ATHases yielded the same (*R*)-product 13 with 58% and 37% ee, respectively. These results support the hypothesis that the DD shielding the biotin-binding vestibule overrides the enantioselectivity preference enforced by the mutation at position S112 or K121.

**Fig. 3:**
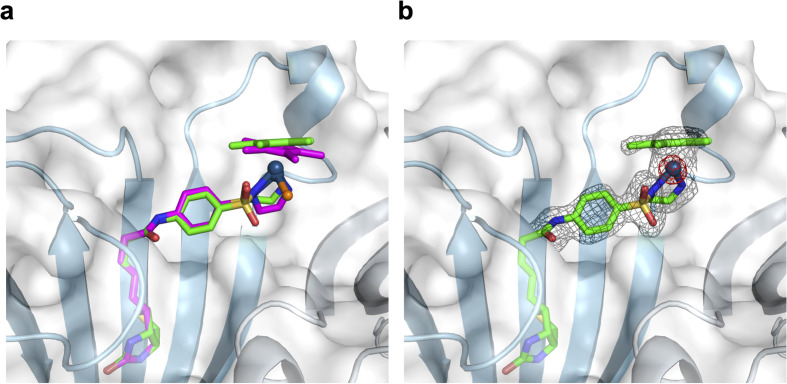
Superposition of crystal structures of ATHase isoforms 1-Cl·Sav S112A (pdb 3pk2) and 1·Sav-SOD S112A (pdb 7b74). The 1-Cl of 3pk2 is represented as a magenta stick model (atoms are color-coded: nitrogen = blue, oxygen = red, carbon = magenta, chloride = orange and sulfur = yellow) with the Ir as a sphere. The 1-Cl of 7b74 is represented as a green stick model (atoms are color-coded: nitrogen = blue, oxygen = red, carbon = green and sulfur = yellow) with the Ir as a sphere. The Sav-SOD S112A protein is represented as a transparent surface and cartoon model. The monomers are color-coded in cyan and gray. Residues 50–83 of the SOD insert could not be resolved in the crystal structure, probably due to disorder. (a) Close-up view of the biotin-binding vestibule, highlighting the superposition of 1-Cl within Sav S112A and Sav-SOD S112A. (b) Close-up view of the biotin-binding vestibule of pdb 7b74 with the modeled 1-Cl in the residual electron density maps from the diffraction measurements. The 2*F*_o_ − *F*_c_ difference map is displayed as a dark grey mesh (1*σ*) and the anomalous electron density map is displayed as a red mesh (8*σ*). The occupancy of the iridium was set to 60%.

To support the latter hypothesis, we collected X-ray diffraction data for single crystals of 1-Cl·Sav-SOD S112A, prepared by co-crystallizing Sav-SOD S112A with 1-Cl over the course of 35 days (see ESI† for details). The structure was solved with a resolution of 1.8 Å. Residual electron density in the biotin-binding vestibule was apparent from the 2*F*_o_–*F*_c_ map and could be modeled with cofactor 1. Further, the iridium was well localized from anomalous dispersion (Fig. 3b). Overlaying the structure of the iridium complex in 1-Cl·Sav S112A (pdb 3pk2)^24^ with the structure of the complex in 1·Sav-SOD S112A (pdb 7b74) enables a direct comparison of the position of the cofactor within the protein scaffolds (Fig. 3a). In both cases, the ATHase bears an Ir_(*S*)_-configuration, and the locations of both cofactors are similar (RMSD = 0.260 Å). Unfortunately, the dimerization domain in 1·Sav-SOD S112A could not be fully resolved due to its high flexibility.^22^ Given the similar location of the cofactors in the Sav and Sav-SOD ATHases, we propose that the enantioselectivity of 1·Sav-SOD S112A is by-and-large influenced by secondary coordination sphere interactions introduced by the dimerization domain. In order to investigate this possibility further, we used QM/MM to identify the most likely transition state involved in the reduction of imine 5 by 1-H·Sav-SOD S112A.

### Quantum mechanics/molecular mechanics calculations

**Fig. 4:**
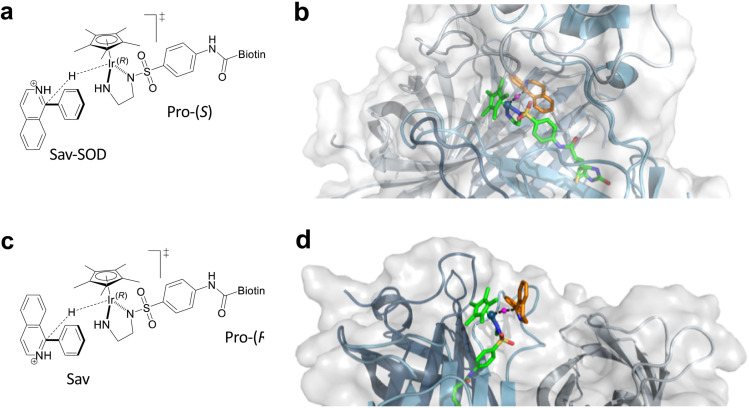
(a) Lowest energy transition state TS3 for the reduction of 5 by Ir_(*R*)_-1-H·Sav-SOD S112A. (b) Modeled transition state TS3 in Ir_(*R*)_-1-H·Sav-SOD S112A. (c) Schematic representation of TS4. (d) Lowest energy transition state TS4 for the reduction of 5 by 1-H·Sav S112A. In (b) and (d), the protein is represented as a cartoon with different colors for each Sav monomer. The solvent-accessible surface of the protein is represented as transparent white. Cofactor 1-H (green) and substrate 5 (orange) are represented as stick models (atoms are color coded: N = blue, O = red, C = green or orange, H = magenta, and S = yellow). The hydride is displayed as a purple sphere.

As noted above, the SOD dimerization domain could not be fully resolved in the crystal structure of 1·Sav-SOD S112A. In order to generate a structure for computational analysis, the crystal structure obtained in this study was supplemented with a sampled structure resulting from molecular dynamics MD simulations in our previous study of the Sav-SOD-based HAMAse.^22^ Only the Ir_(*R*)_ configuration of 1-H was considered in Sav-SOD S112A. This is justified since, for the Ir_(*S*)_-1-H, the hydride cannot be delivered to the prochiral imine as it points towards the bottom of the biotin-binding vestibule. Several initial poses were examined for binding of imine 5 in the ATHase, and all of these converged to four possible transition states (Fig. 4 and S3† and Table 2). Of these possible transition states, TS1 and TS3 lead to (*S*)-11, whereas TS2 and TS4 lead to (*R*)-11. The calculations revealed that the TS3 conformation has the lowest energy for 1-H·Sav-SOD S112A. We also performed analogous calculations for 1-H·Sav S112A, and in this case, the cofactor is more solvent-exposed, so that both Ir-configurations of 1-H are possible. This leads to four extra possible transition states in addition to TSi–TSiv (Fig. S3† and Table 2). The most favorable transition state for the conversion of 5 by 1-H·Sav S112A ATHase is TS4, which preferentially affords the (*R*)-product 11. Interestingly, in the absence of the SOD dimerization domain, substrate 5 can approach in such a way that the bulky phenyl group is oriented towards the solvent, thereby reducing steric hindrance and making this particular conformation more stable than those found in the other transition states. In contrast, the presence of the SOD dimerization domain in 1-H·Sav-SOD S112A causes steric hindrance and destabilization of the substrate in TS4. For both 1-H·Sav-SOD S112A and 1-H·Sav S112A, the transition state is positively charged, and the presence of the lysine at position 121 may therefore be destabilizing. On the other hand, in the case of the Sav-SOD ATHase, the SOD dimerization domain may stabilize the transition state, given that it contains many negatively-charged amino acid residues. Therefore, the negatively skewed electric field around the catalyst may result in a lowered transition state energy, which may lead to increased reaction rates.

**Table 2:** Computed transition state energy differences for the reduction of substrate 5 by 1-H·Sav-SOD S112A or 1-H·Sav S112A

Transition state	Ir–H configuration	Configuration of product 11	Energy of transition state (kcal mol^−1^)	
			Sav-SOD	Sav
TS1	Ir_(*R*)_	(*S*)	27.13	13.16
TS2	Ir_(*R*)_	(*R*)	32.47	25.21
TS3	Ir_(*R*)_	(*S*)	**0.00**	17.29
TS4	Ir_(*R*)_	(*R*)	4.65	**0.00**
TSi	Ir_(*S*)_	(*R*)	—	12.17
TSii	Ir_(*S*)_	(*S*)	—	1.46
TSiii	Ir_(*S*)_	(*R*)	—	21.16
TSiv	Ir_(*S*)_	(*S*)	—	14.82

### Kinetics of imine reduction using PDQ

The marked differences in catalytic behavior between the Sav and Sav-SOD ATHases for substrate 5 warranted further kinetic investigation. Thus, we analysed the Michaelis–Menten saturation kinetics in 300 mM MOPS buffer (pH = 6.0) with fixed concentrations of sodium formate (2 M). As observed previously for Sav-based ATHases,^13,25,35^ we observed the imine substrate saturation kinetic profiles. A summary of these data is presented in Table 3 and Fig. S4–S5.†

**Table 3:** Results from the saturation kinetic experiments with the substrate PDQ 5. The PDQ-dependent kinetic profiles were determined at 25 and 37 °C with a total reaction volume of 400 μL and a fixed concentration for streptavidin (Sav) or Sav-SOD (30 μM), cofactor (15 μM), MOPS (300 mM) and sodium formate (2 M). The reaction was initiated upon addition of the substrate stock solution yielding a final substrate concentration ranging from 5–50 mM. Aliquots (50 μL) were collected at 10, 20, 30, 45, 60 and 90 min time intervals, quenched, and analyzed by GC-MS. Full details are collected in the ESI†

	ATHase	*k*_cat_ (min^−1^)	*K*_M_ (mM)	*k*_cat_/*K*_M_ (min^−1^ mM^−1^)
25 °C	1-H·Sav S112A	2.13	6.02	0.35
	1-H·Sav K121A	3.93	9.51	0.41
	1-H·Sav-SOD S112A	11.71	14.19	0.83
	1-H·Sav-SOD K112A	3.61	4.10	0.88
37 °C	1-H·Sav S112A	6.44	15.15	0.43
	1-H·Sav K121A	7.88	10.64	0.74
	1-H·Sav-SOD S112A	13.83	38.96	0.35
	1-H·Sav-SOD K112A	16.00	29.20	0.55

Substrate-dependent ATHase activity profiles were determined at 25 °C and 37 °C. At 37 °C, the Sav-SOD ATHase systems exhibited approximately double the *k*_cat_ compared to their Sav-based counterparts. This is partly compensated by the respective Michaelis constant (*K*_M_) for the Sav-SOD ATHases. At 25 °C, the catalytic efficiency of 1·Sav-SOD-derived ArMs is approximately twice as large as that of 1·Sav-derived ArMs. Strikingly, however, this trend is (partially) reversed at 37 °C, whereby 1·Sav-derived ArMs are more efficient.

We next sought to investigate whether the rate acceleration observed for the Sav-SOD ATHases results from greater efficiency in the Ir–H complex formation or the subsequent imine reduction step. We collected additional ATHase saturation kinetic profiles with varying concentrations of sodium formate from 0.25 to 4.0 M. We performed these experiments at 25 °C with an initial concentration of 50 mM for PDQ 5 to ensure that all ATHases were operating above their respective *K*_M_ values. The kinetic results are collected in Fig. S6.† We observe that the *K*_M_ for sodium formate is significantly lower for the Sav-SOD ATHases. We find *K*_M_ values for sodium formate of approximately 0.78 M *vs.* >2.74 M for 1-H·Sav-SOD S112A *vs.*1-H·Sav S112A, respectively, and approximately 0.67 M *vs.* >4.85 M for 1-H·Sav-SOD K121A *vs.*1-H·Sav K121A, respectively. We hypothesize that this lowering of the *K*_M_ for sodium formate might be a result of interactions with the DD, facilitating the formation of the active Ir–H, 1-H. Furthermore, in the case of 1-H·Sav S112A, we found that the activity decreases with increasing formate concentration, although this inhibition is not observed for 1-H·Sav-SOD S112A. The trends in *k*_cat_ are less clear. In the case of 1-H·Sav S112A, substrate inhibition is observed. Due to the limited solubility of sodium formate in aqueous solution, it was not possible to reach the saturation concentrations for either Sav or Sav-SOD. However, the catalytic efficiency for the formation of the 1-H·ATHases (*k*_cat_/*K*_M_) was found to be higher for the Sav-SOD ATHases (17.71 *vs.* 1.87 M^−1^ min^−1^ and 6.70 *vs.* 2.83 M^−1^ min^−1^ for Sav-SOD S112A *vs.* Sav S112A and Sav-SOD K121A *vs.* Sav K121A, respectively – see Table S4†). The imine reduction reaction (*i.e.*, the iridium-hydride consuming reaction) was found to be 5.5-fold faster for 1-H·Sav-SOD S112A than for 1-H·Sav S112A (11.71 *vs.* 2.13 M^−1^ min^−1^) and about the same (3.61 *vs.* 3.93 M^−1^ min^−1^) for 1-H·Sav-SOD K121A and 1-H·Sav K121A. Therefore, we propose that the increased yields observed for the Sav-SOD constructs may be in part due to the more efficient formation of the hydride catalyst.

## Conclusion

In summary, we have evaluated the chimeric streptavidin Sav-SOD as a scaffold for an ATHase incorporating [Cp*Ir(biot-*p*-L)Cl] (1-Cl) as a cofactor. The dimerization domain presents a secondary coordination environment that is inherently hydrophobic and capable of shielding tethered biotinylated cofactors from the aqueous milieu, akin to natural enzymatic systems. Our investigation of Sav-SOD based ATHases has revealed that, depending on the nature of the substrate, the dimerization domain can alter the catalytic efficiency and selectivity of iridium-catalyzed imine transfer hydrogenation reactions. In particular, in the case of the substrate 1-phenyl-3,4-dihydroisoquinoline (5), 1·Sav-SOD and 1·Sav ATHases exhibit opposite, albeit modest, enantioselectivities. Single-crystal X-ray diffraction data suggest that the configuration of the cofactor in both systems, 1-H·Sav S112A and 1-H·Sav-SOD S112A, is the same, although both ATHases afford opposite enantiomers of amine 11. QM/MM calculations suggest that the DD forces the substrate PDQ 5 to preferentially present its pro-(*S*) face to the Ir_(*R*)_ cofactor in the transition states of 1-H·Sav-SOD S112A, providing a rationale for their opposite enantioselectivity. Further catalytic experiments will need to be performed in order to fully characterize the reactivity of the Sav-SOD ATHases.

## Conflicts of interest

There are no conflicts to declare.

## Supplementary Material

FD-244-D3FD00034F-s001
