# Supplementary material for: Spiers Memorial Lecture: Shielding the active site: a streptavidin superoxide-dismutase chimera as a host protein for asymmetric transfer hydrogenation
Source: Faraday Discuss. 2023 Mar 16;244:9–20. doi: 10.1039/d3fd00034f (PMC10416703; doi:10.1039/d3fd00034f)
Supplement: FD-244-D3FD00034F-s001 [file FD-244-D3FD00034F-s001.pdf]

Electronic Supplementary Material (ESI) for Faraday Discussions.
This journal is © The Royal Society of Chemistry 2023

Supporting Information

Shielding the Active Site: A Chimeric Streptavidin Superoxide-Dismutase Chimera as Host Protein for Asymmetric Transfer Hydrogenation

Nico V. Igareta,[a,c] Ryo Tachibana,[a] Daniel C. Spiess,[a] Ryan L. Peterson,[b,c*] Thomas R. Ward[a,c*]

[a]Department of Chemistry, University of Basel, Mattenstrasse 24a, BPR 1096, CH-4058, Basel, Switzerland

[b]Current address: Texas State University, Department of Chemistry and Biochemistry 601 University Dr., San Marcos, TX 78666

[c]National Center of Competence in Research (NCCR) "Molecular Systems Engineering", 4058 Basel, Switzerland

correspondence: thomas.ward@unibas.ch, rlp139@txstate.edu

Table of Contents

GENERAL INFORMATION. 2

CLONING AND EXPRESSION OF SAV AND SAV-SOD MUTANTS. 2

ATHASE KINETIC MEASUREMENTS. 3

PROTEIN ESI-MS DETERMINATION. 3

SINGLE-CRYSTAL X-RAY DIFFRACTION ANALYSIS OF [CP*IR(BIOT-P-L)CL]·SAV-SOD-S112A. 4

TRANSFER HYDROGENATION REACTIONS FOR CHIRAL PRODUCT YIELD. 6

CHIRAL AMINE ANALYSIS. 6

KINETIC MEASUREMENTS ANALYSIS. 12

ITC ANALYSIS. 14

QM/MM CALCULATIONS. 15

REFERENCES 16

General information. Chemicals were purchased from Sigma Aldrich, Acros Organics, Alfa Aesar or Fluorochem and used without further purification. Water used for molecular biology and in the catalytic reactions was purified by a Milli-Q Advantage system. All catalytic reactions were carried out with non-degassed solvents under air. Temperature was maintained using Thermowatch-controlled heating blocks. Gas chromatography–mass spectrometry (GC-MS) analysis was run on a Shimadzu GCMS-QP2020. A normal phase HPLC instrument from Agilent with a Chiralpak IB column (5 μm, 4.6 mm × 250 mm, Daicel) and a UPC2 system (Waters) was used to analyze the samples. High-resolution mass spectrometry (HR-MS) was performed on a Bruker maXis II QTOF ESI mass spectrometer coupled to a Shimadzu LC. The transfer hydrogenation co-factor [Cp*Ir(biot-*p*-L)Cl] was synthesized as previously reported.[1] Imine reduction yields were determined using an internal standard or substrate to production response factor. Chiral amine retention times and enantiomeric excess (*ee*) yields were determined using authentic and racemic standards. Molecular biology reagents were purchased from New England Biolabs (NEB), Integrated DNA Technologies (IDT), and Macherey-Nagel using accompanying protocols. Protein concentrations were determined by the calculated A_{280} molar absorptivity coefficients using the ProtParam tool (ExPASy) of 41940 M^{-1} cm^{-1} and 43430 M^{-1} cm^{-1} for wild type Sav and Sav-SOD respectively.

Cloning and expression of Sav and Sav-SOD mutants. The Sav-SOD chimera gene was synthesized and cloned into the NcoI and EagII restriction sites of the pET28 vector by Gene Universal Inc. (Newark, DE). Site directed mutagenesis was achieved using the primers listed in Table S1, followed by BsaI/DpnI digestion and ligated using T4 ligase. Mutations were verified by Sanger sequencing performed by Microsynth

(Balgach, Switzerland). The expression and purification of Sav and Sav-SOD proteins were achieved using auto induction media (ZYP-5052) followed by cell lysis and purification using iminobiotin agarose affinity chromatography as previously described.[2]

Table S1 Primers used for mutagenesis.

Oligo	Sequence	
oRP222A	CATAT*ggtctc*TTTGCTTCAGTAGTGCCGGCGGTCAGCAGCCACTGGGTATT	S112A
oRP225S	CATAT*ggtctc*TTTGCTTCAGTAGTGCCGCTGGTCAGCAGCCACTGGGTATT	S112S
oRP226K	TATAA*ggtctc*AGCAAATGCCTGGAAAAGCACCCTGGT	K121K
oRP231A	TATAA*ggtctc*AGCAAATGCCTGGGCAAGCACCCTGGT	K121A

ITC analysis. Biotin binding affinities were measured using a Microcal VP-ITC as previously described by Stayton and Coworkers.[3] A 10 μM solution of [Cp*Ir(biot-*p*-L)Cl] in 50 mM sodium phosphate buffer containing 100 mM sodium chloride at pH = 7.75 with 2.5 % DMSO was titrated with 5 μL injections of Sav-S112A or Sav-SOD-S112A solutions (100 μM, 50 mM sodium phosphate, 100 mM sodium chloride, 2.5% DMSO, pH = 7.75). The reference cell contained the same buffer as the protein and cofactor solution. Measurements were performed at 25 °C. The [Cp*Ir(biot-*p*-L)Cl] binding constant K_a, enthalpy *h*, and number of binding sites at each temperature were calculated using ITC data analysis origin software (MicroCal).

*Table S2 Thermodynamic parameters for [Cp*Ir(biot-p-L)Cl]* ***1****-Cl binding to Sav and Sav-SOD mutant proteins determined in this work. All data were collected at 25 °C. Standard deviations are given in parentheses.*

Protein	K_a (M^{-1})	ΔG° (kcal/mol)	ΔH° (Kcal/mol)	TΔS° (Kcal/mol)
Sav-S112A	1.15(0.20) × 10^8	−14.8	−14.5(0.6)	−0.3
Sav-SOD-S112A	1.27(0.31) × 10^8	−13.8	−13.6(0.9)	−0.2

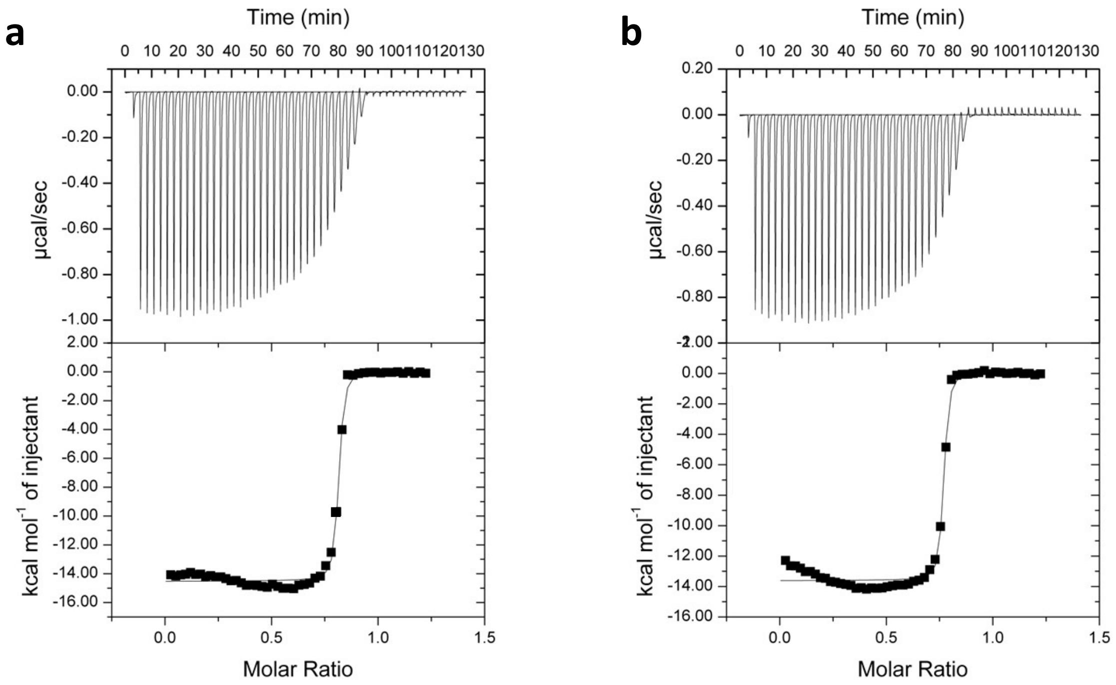

Figure S1: ITC analysis of **a** [Cp*Ir(biot-p-L)Cl] · Sav-S112A and **b** [Cp*Ir(biot-p-L)Cl] · Sav-SOD-S112A.

Protein ESI-MS determination. Proteins were dissolved in Milli-Q water, 1% formic acid (pH = 2.5) with a final concentration of 0.2 mg/mL and clarified by centrifugation. A HPLC (Shimadzu, equipped with a Jupiter® 5 μm C4 300 Å)-ESI QTof (Bruker Daltonics, ESI MaxisII QTof MS) system was used for data collection . The ESI-QTof mass spectrometer was calibrated with ESI-Tof TuneMix (Agilent). The charge envelope from 2800–4500 m/z for the Sav-SOD and from 2500–4000 m/z for the Sav, was deconvoluted using the Compass Data Analysis software (Bruker Daltonics) with

the Maximum Entropy setup. For samples with [Cp*Ir(biot-*p*-L)Cl], the proteins were dissolved in Mili-Q water with a concentration of 0.5 mg/ml and 4 equiv. of the cofactor. After 15 min, the samples were diluted with Mili-Q water, 1% of formic acid (pH = 2.5) to a final protein concentration of 0.2 mg/ml and clarified by centrifugation. The charge envelope from 2800–4500 m/z was deconvoluted using the Compass DataAnalysis software (Bruker Daltonics) with the Maximum Entropy setup.

Table S3. Predicted and observed mass for Sav-SOD and Sav-SOD ATHases.

Mutant	Mass calculated (Da)*	Mass observed (Da)
SavSOD-K121A	79699.2	79733.5
**1**·Sav-SOD-K121A (0.25 eq **1**)	80466.4	80501.0
**1**·Sav-SOD-K121A (0.5 eq **1**)	81233.6	81268.4
**1**·Sav-SOD-K121A (0.75 eq **1**)	82000.9	82005.2
**1**·Sav-SOD-K121A (1 eq **1**)	82768.1	No signal detected
SavSOD-S112A	79862.9	79869.0
**1**·Sav-SOD-S112A (0.25 eq **1**)	80630.1	80636.8
**1**·Sav-SOD-S112A (0.5 eq **1**)	81397.3	81435.5
**1**·Sav-SOD-S112A (0.75 eq **1**)	82073.5	No signal detected
**1**·Sav-SOD-S112A (1 eq **1**)	82840.8	No signal detected

** Masses calculated for tetrameric Sav-SOD.*

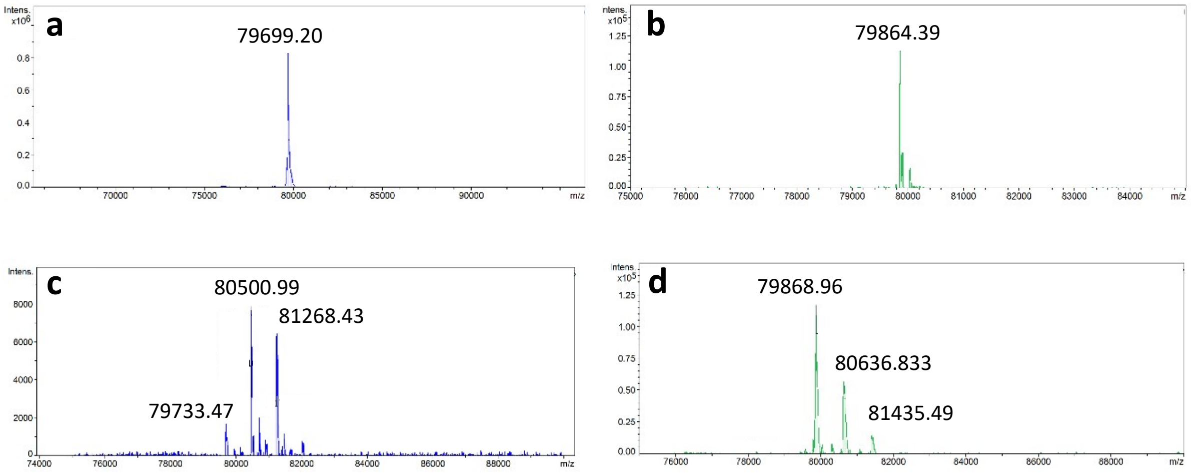

Figure S2: Exact mass of Sav-SOD chimeras and ArMs. ***a*** *Deconvoluted mass spectra of Sav-SOD-K121A chimera.* ***b*** *Deconvoluted mass spectra of Sav-SOD-S112A.* ***c*** *deconvoluted mass spectra of* ***1*** *· Sav-SOD-K121A chimera.* ***d*** *Deconvoluted mass spectra of* ***1*** *· Sav-SOD-S112A. Calculated and determined masses are for the chloride-free [Cp*Ir(biot-p-L)]* ***1*** *· ATHases are collected in Table S3. Panels* ***c-d*** *represents MS spectra collected at a 0.5 eq loading stoichiometry.*

QM/MM calculations. In order to calculate the structure of Sav-SOD ATHases, we first estimated by MD simulation the structure of the loops –i.e. the dimerization domain– whose structure could not be resolved from our X-ray crystallography data sets. The sampled loop segment was combined with the crystal structure of streptavidin (pdb: 7ALX, tetramer)[4] to form the initial Sav-SOD structure used for structural optimization. Only two Sav-SOD monomers, which make up the biotin-binding vestibule, were used throughout the computation. A proton at pH 7.4 was assigned to each amino acid residue using the PROPKA server.[6] Here, the [Cp*Ir(biot-*p*-L)H] catalyst structure was appended so that the coordinates of its biotin moiety were the identical to the X-ray structure. Then the structure of the substrate was also appended. Considering the C2-relationship between the two adjacent biotin-binding sites, the cofactor was anchored only in one of two biotin-binding sites that make up the biotin-binding vestibule. In the structural optimization, only the substrate PDQ **5**, catalyst (except for the bicyclic urea moiety of biotin), the SOD loop, and other nearby

amino acid residues (111-122) were allowed to relax, while the coordinates of other parts were frozen.

Calculations were performed at the level of ONIOM(PBEPBE:UFF). The basis set was Def2TZVPP for Ir atoms and 6-31G(d) for other atoms. The high layer included only substrates and cofactor (except for the fused urea moiety of biotin). The atomic charges for the cofactor and the substrate were computed at the same level as the high layer. Electronic embedding was used in all calculations except for the transition-state structure optimization.

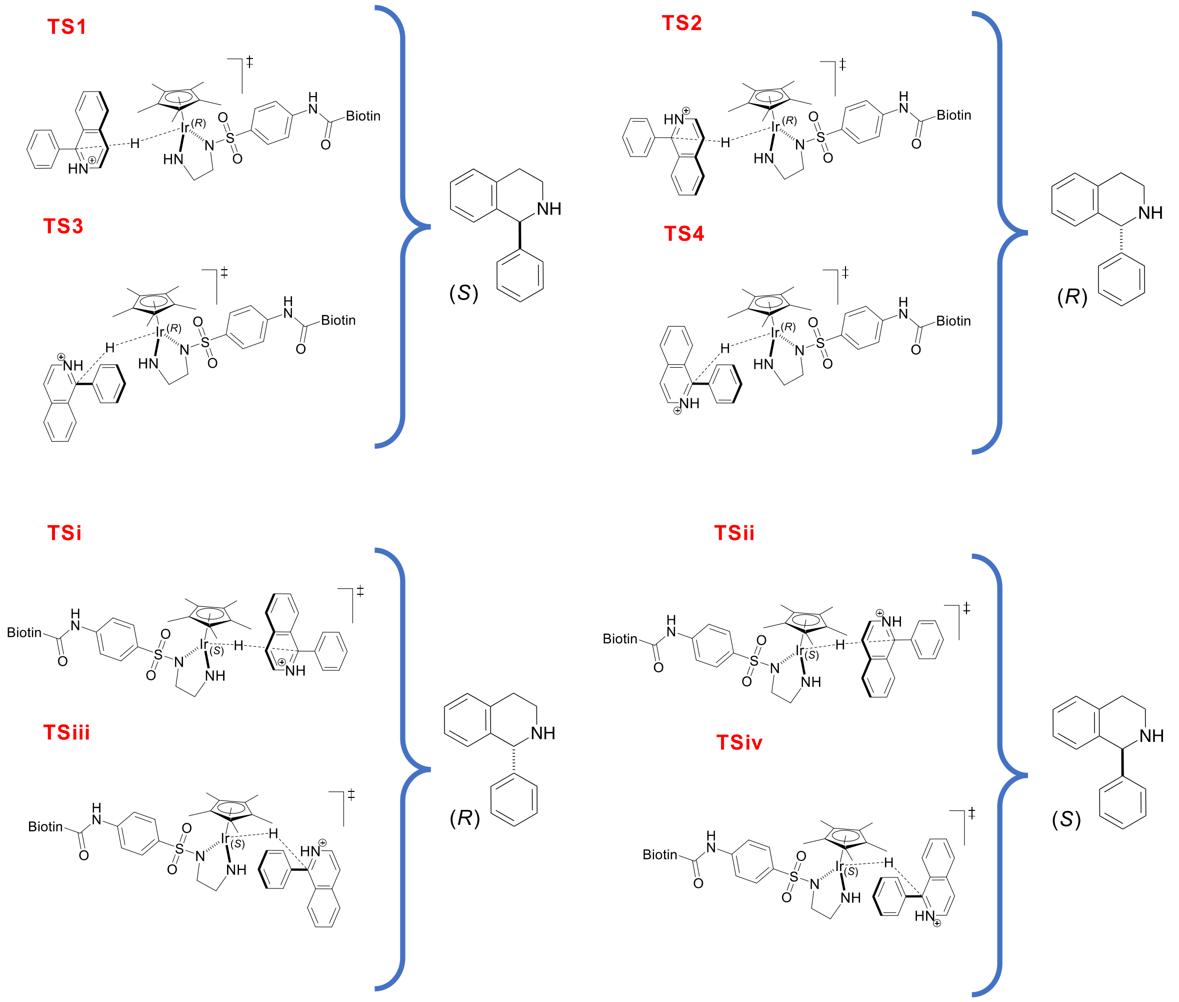

*Figure S3. Computed transition states for the ATHases with the [Cp*Ir(biot p L)H] **1**-H cofactor and pro-chiral PDQ (**5**) substrate.*

ATHase Kinetic Measurements. The 1-phenyl-3,4-dihydroisoquinoline (PDQ)-**5** dependent kinetic profiles were determined at 25 and 37 °C, with a total reaction volume of 400 μL. The final concentrations of the reactions were as follows: streptavidin (Sav) or Sav-SOD (30 μM biotin binding sites, corresponding to 7.5 μM of the homotetramer), cofactor (15 μM), MOPS buffer (300 mM) and sodium formate (2 M). The *in situ* assembly of the ATHase was achieved by adding a solution of [Cp*Ir(biot-*p*-L)Cl] in *N*,*N*-dimethylformamide (DMF) (4 mM) to a solution of the scaffold protein for 10 min at room temperature. The volume was adjusted to 160 μL with Milli-Q water and 200 μL of a of MOPS (300 mM) and sodium formate (2 M) buffer solution was added. The reaction was initiated upon the addition of a 10× substrate stock solution (40 μL) yielding a final substrate concentration ranging from 5 – 50 mM. An aliquot (50 μL) of the reaction was collected at 10, 20, 30, 45, 60, 90 min time intervals and quenched by the addition of 40 μL of freshly prepared 0.2 M reduced glutathione. Reaction aliquots were diluted with Milli-Q water (200 μL) and made basic with the addition of 50 μL of 20% w/v aqueous NaOH. The organic products were extracted with dichloromethane (600 μL), dried with anhydrous sodium sulfate and analyzed by GC-MS.

Formate-dependent reaction kinetics were performed using the same procedure described above using 200 μL of MOPS (300 mM) buffer stock solutions (pH = 6.0) with varying sodium formate concentrations. The reaction was initiated upon the addition of 40 μL 0.5 M PDQ.

Kinetic measurements analysis. Kinetic measurements of the reduction of PDQ **5** were analyzed using a Shimadzu, GCMS-QP2020 equipped with an Agilent HP1-1MS column (length: 30 m; Diameter: 0.25 mm; Film: 0.25 μM). A single acquisition mode

was used with m/z range of 175 – 180. The product was quantified based on the relative product peak area and the substrate area using a response factor of 1.19.

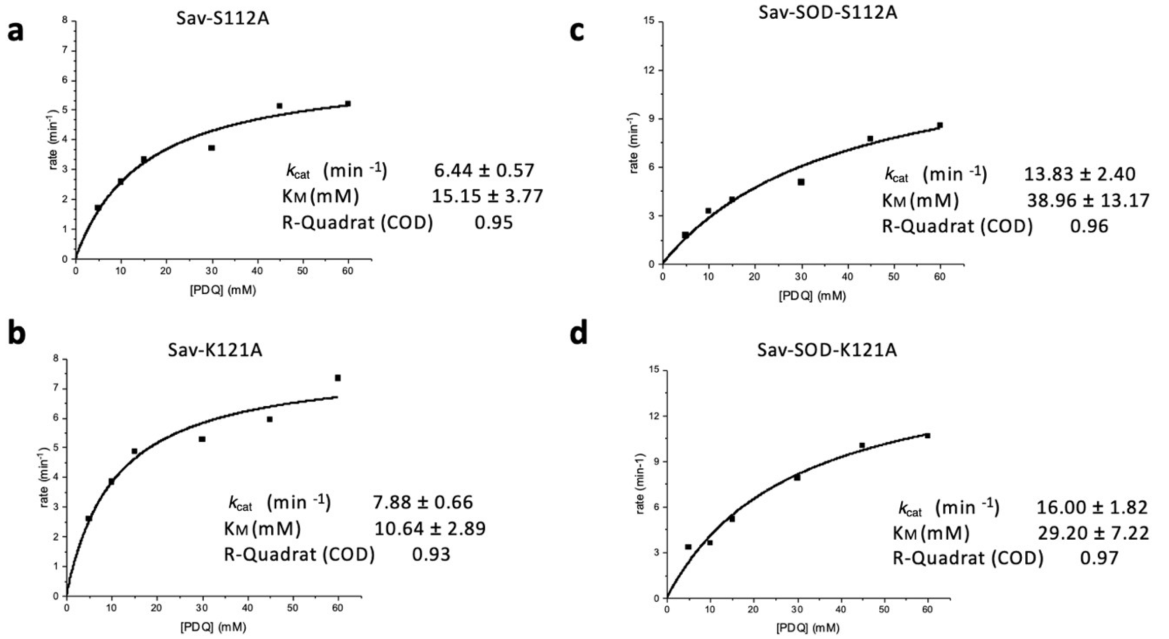

*Figure S4 Michaelis-Menten kinetic profiles for the reduction of **5** (PDQ) in 2 M sodium formate buffer pH at 37 °C.*

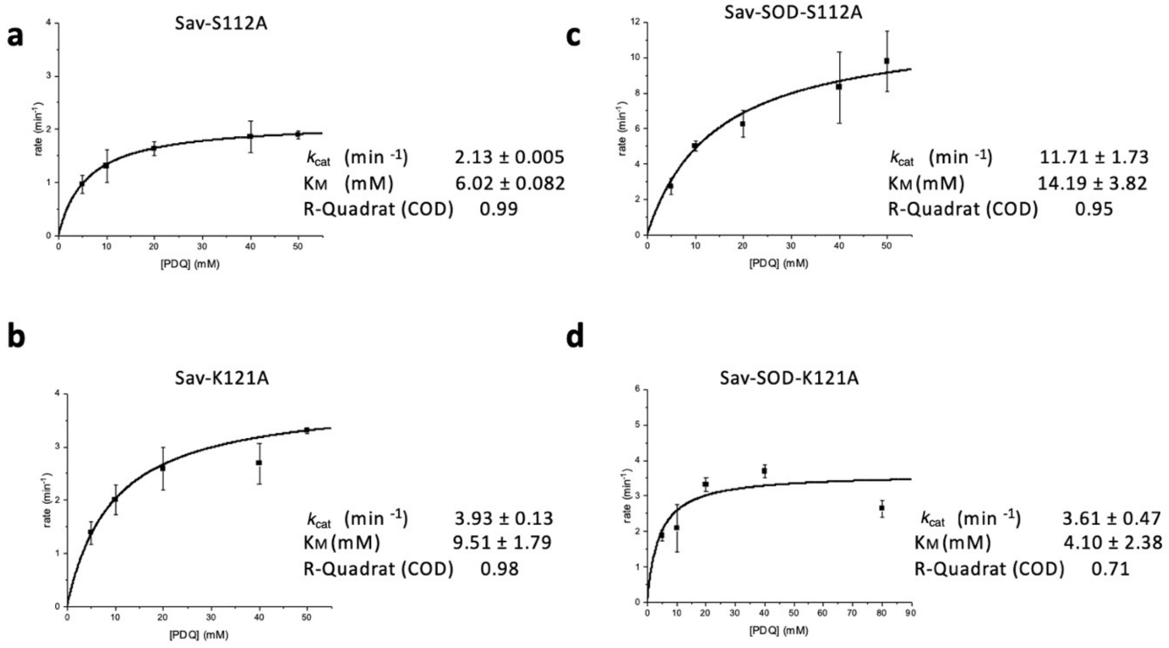

*Figure S5 Michaelis-Menten kinetic profiles for the reduction of **5** (PDQ) in 2 M sodium formate buffer pH at 25 °C*

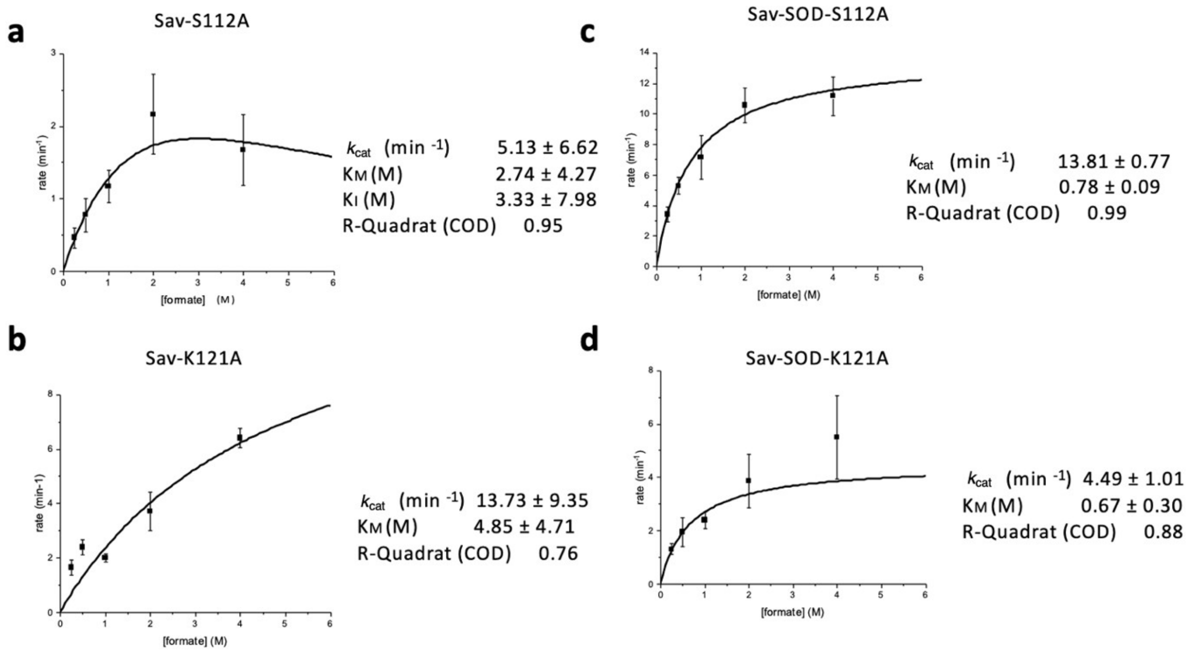

*Figure S6 Michaelis-Menten kinetic profiles for the reduction of **5** (PDQ) with varying concentrations of sodium formate at 25 °C.*

*Table S4. Collected kinetic parameters for the reduction of PDQ (**5**) (50 mM) at varying formate concentrations.*

Temp	ATHase	k_{cat} (min^{-1})	K_M (mM)	k_{cat}/K_M (min^{-1} mM^{-1})
25 °C	Sav S112A	5.13	2.74	1.87
	Sav K121A	13.73	4.85	2.83
	Sav-SOD S112A	13.81	0.78	17.71
	Sav-SOD K121A	4.49	0.67	6.70

Single-crystal X-ray diffraction analysis of [Cp*Ir(biot-p-L)Cl] · Sav-SOD-S112A. Protein crystals were obtained using a sitting drop vapor diffusion method. The lyophilized protein mutant (Sav-SOD-S112A) was dissolved in a 20 mM Tris-HCl solution (pH = 7.0) to a concentration of 2.5 mg/mL. A 10 mM solution of [Cp*Ir(biot-*p*-L)Cl] in dimethylsulfoxide (DMSO) (5 µL) was added to the solution and the mixture was incubated at room temperature overnight. After concentration to 25 mg/mL by ultracentrifugation (Amicon® ultra centrifugal filters, Merck; cut-off of 10 kDa), crystals were grown by sitting drop vapor diffusion using 20 %w/v PEG 3350,

0.2 M KF as crystallization buffer. Crystals grew after 30 days and were flash frozen in liquid nitrogen without further cryoprotection. Protein crystal diffraction data was collected at 100 K at the Swiss Light Source beamline PXI with a wavelength of 1.0 Å and analyzed with CCP4i2 Suite.[5] Crystal indexing, integration and scaling were carried out with XDS and reflections were merged with AIMLESS. PHASER MR was used to solve the structure by molecular replacement with PDB 2BC3 as input model. For structure modeling, water picking and electron density visualization the software COOT was used. Amino acid residues 1-14, 51-85 and 172-196 are not resolved in the electron density, presumably due to disorder.

Residual electron density was observed in the biotin-binding pocket and in the biotin vestibule from the $F_o - F_c$ map. Furthermore, anomalous scattering was observed in the vestibule. Modeling of cofactor [Cp*Ir(biot-*p*-L)Cl] **1** into the electron density placed the iridium in the position of the anomalous-density (Figure S7).

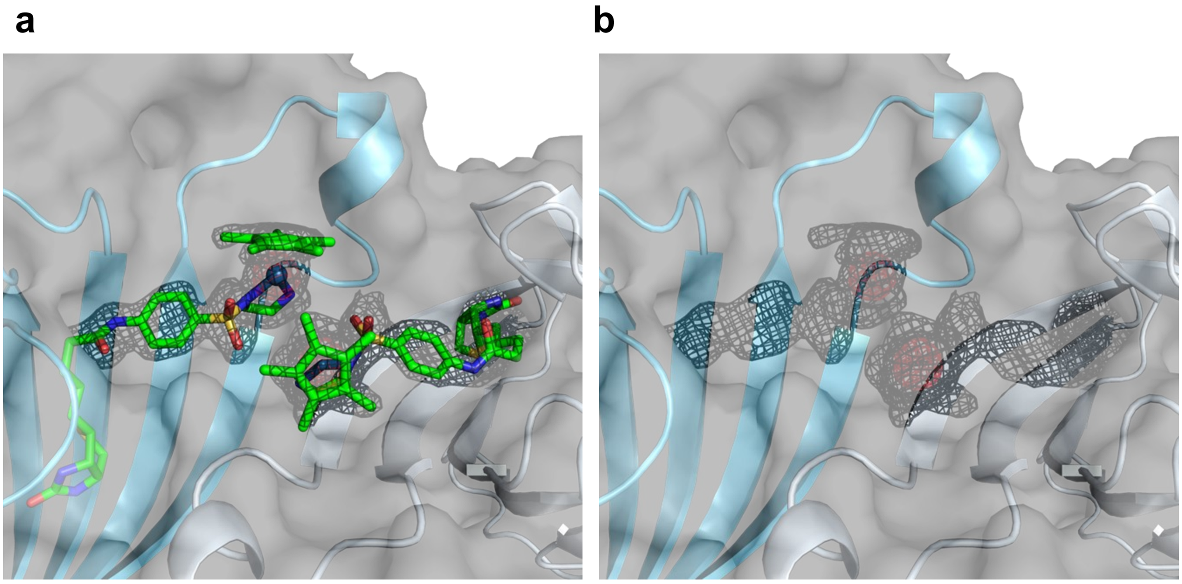

*Figure S7 Single-crystal X-ray diffraction structure of [Cp*Ir(biot-p-L)Cl]* ***1****-Cl · Sav-SOD-S112A highlighting the biotin-binding site of two facing monomers of Sav-SOD-S112A with (****a****) and without (****b****) [Cp*Ir(biot-p-L)Cl]* ***1****-Cl modeled in, –cofactor displayed as color-coded stick with iridium represented*

as a blue sphere. The $2F_o-F_c$ map is displayed as a grey mesh with 1.0 σ. The anomalous density obtained from the measurement is shown in red (5.0 σ).

Table S5 Data processing and crystal structure refinement statistics.

**Data collection**	[Cp*Ir(biot-*p*-L)Cl] · Sav-SOD S112A
Space group	P 1 2_1 1
Cell dimension	
a, b, c (Å)	57.28, 57.31, 88.03
α, β, γ (°)	90, 94.62, 90
Resolution (Å)	47.98–1.0
R_{merge}	15.9 (127.0)
I/σ(I)	9.2 (1.56)
$CC_{1/2}$	1.00 (0.55)
Completeness (%)	98.3 (98.0)
**Refinement**	
Resolution (Å)	47.98–1.85
No. reflections	15314
R_{work}/R_{free}	20.2 / 22.6

Transfer hydrogenation protocol. To test the hydrogenation of prochiral imines with the Sav-SOD-based ATHases, reactions were run for 24 hours at 37 °C. The following stock solutions were prepared: (i) Substrates (400–500 mM in DMSO, ethyl acetate or H_2O); (ii) [Cp*Ir(biot-*p*-L)Cl] (4 mM in DMF); (iii) protein (400 μM in H_2O) and (iv) MOPS-formate buffer (600 mM MOPS with 4 M sodium formate, pH = 6.0). These solutions were added in the following order and final concentrations to an HPLC glass vial (total reaction volume 200 μL): 1) [Cp*Ir(biot-*p*-L)Cl] **1** 50 μM; 2) Sav 100 μM; 3) MOPS-formate buffer 300 mM and 2 M; 4) substrate 10 mM. Solutions 1)-3) were added sequentially, and incubated for 10 min at room temperature. The reaction was initiated upon the addition of the substrate solution 4) and run in an Eppendorf

thermomixer (37 °C, 1000 rpm) for 24 h. The reactions were quenched by the addition of 20 % w/v NaOH (50 μL), at which point the internal standard was added to the reaction vessel and extracted with 600 μL ethyl acetate. The organic phase was collected and analyzed.

Quantification of product reduction yields and enantioselectivity. The conversion and enantiomeric excess were determined either using an Agilent HPLC or an ACQUITY UPC2 Waters system using the chiral columns and separation conditions detailed below.

Analysis of imine substrate **2** and corresponding amine products (*S*)-**8** and (*R*)-**8** was performed with a Chiralpak IC column (5 μm, 4.6 mm × 250 mm, Daicel) using a constant flow rate (2.5 m/min). Biphenyl was used as an internal standard. The following program was set-up used for analysis: 98% CO_2 and 2% IPA (0.1% DEA) from 0–4 min; linear increase to 80% CO_2 and 20% IPA (0.1% DEA) from 4–6 min; linear increase to 98% CO_2 and 2% IPA (0.1% DEA) from 6–8 min; maintain at 98% CO_2 and 2% IPA (0.1% DEA) from 8–10 min. Retention times: 2.3 min for biphenyl, 3.7 min for (*R*)-**8**, 3.9 min for (*S*)-**8**, 7.3 min for **2**. The compounds were quantified based on the relative product areas and internal standard area (absorbance at 254 nm) using a response factor of 0.5023.

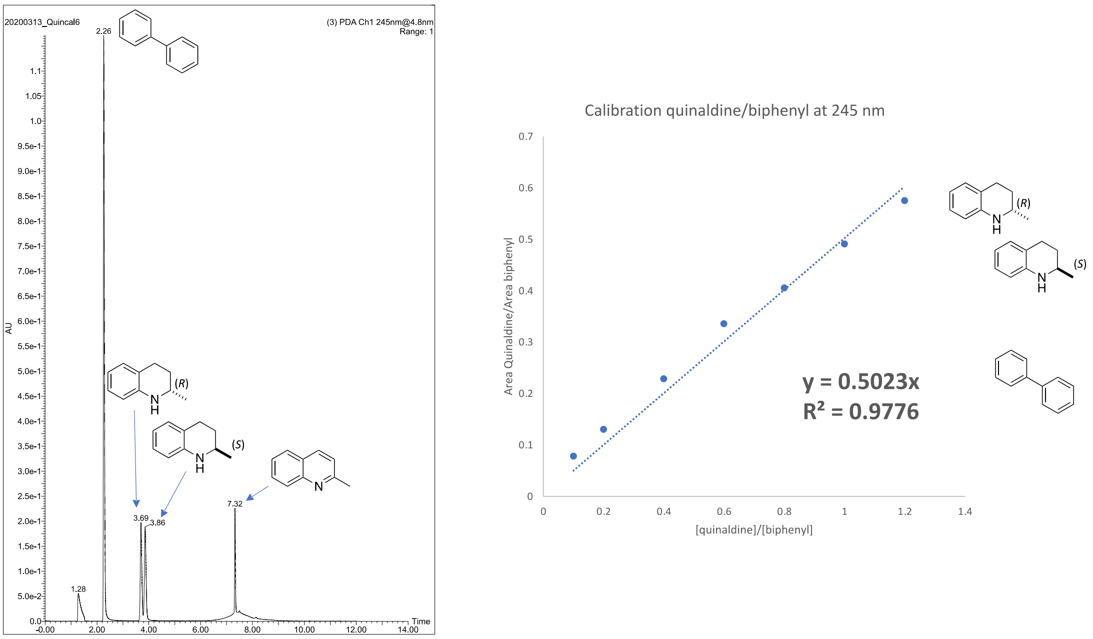

Figure S8 Chromatogram and calibration curve for substrate ***2*** *and corresponding product* ***8****. Left: UPC2 chromatogram for the imine* ***2****, amine (R)- and (S)-****8*** *and the internal standard biphenyl at 10 mM concentrations. Right: The calibration curve for (R)- and (S)-****8*** *versus internal standard. The calibration curve was made in a range from 0.5 mM to 5 mM.*

Analysis of **3**, (*S*)-**9** and (*R*)-**9** was performed with a Chiralpak IC column (5 µm, 4.6 mm × 250 mm, Daicel) using constant 90% CO_2 and 10% IPA (0.1% DEA) at a constant flow rate (2.5 mL/min) on a Waters UPC2. 1-Methoxynaphthalene was used as an internal standard. The retention times were 2.1 min for 1-methoxynaphthalene, 5.6 min for **3**, 9.3 min for (*R*)-**9**, 10.2 min for (*S*)-**9**. The compounds were quantified based on the relative product peak areas and the internal standard area (absorbance at 210 nm) using a response factor of 0.1213.

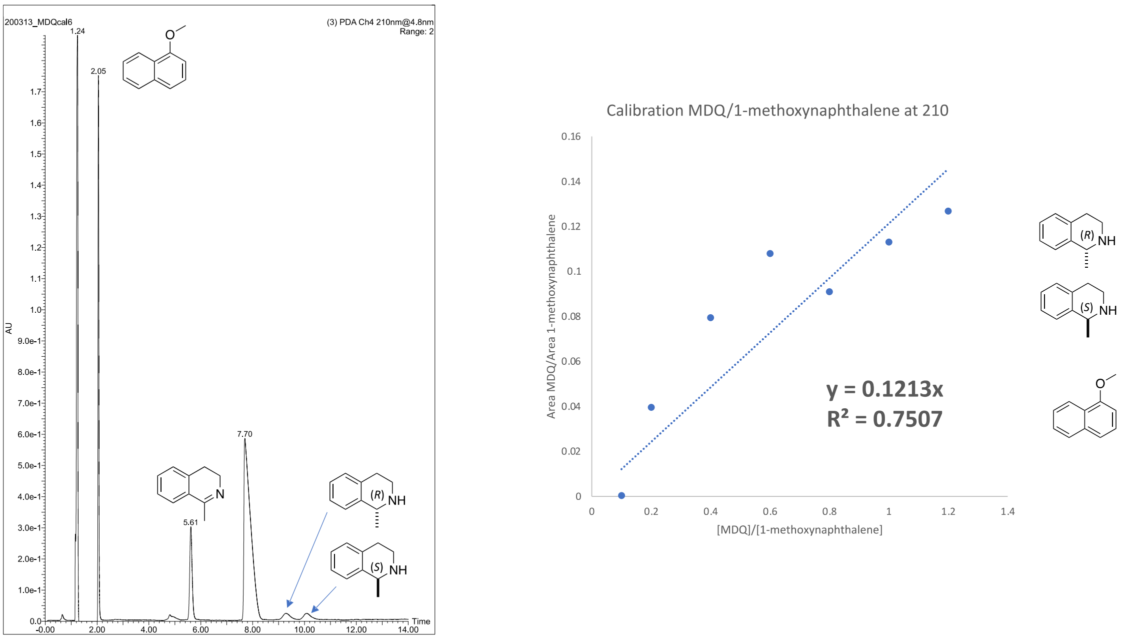

*Figure S9 Chromatogram and calibration curve for substrate **3**. Left: UPC2 chromatogram for the imine **3**, amine* (R)*-**9*** (S)*-**9**, and the internal standard 1-methoxynaphthalene in 10 mM concentrations. Right: The calibration curve for* (R)*- and* (S)*-**9** versusthe internal standard 1-methoxynaphthalene using the UPC2 method. The calibration was made in a range from 0.5 mM to 5 mM.*

Analysis of **4**, (*S*)-**10** and (*R*)-**10** was performed using a Chiralpak ID column (5 µm, 4.6 mm × 250 mm, Daicel) at a constant flow rate (2.5 mL/min) UPC2. Biphenyl was used as an internal standard. The program was set up as follows: 85% CO_2 and 15% IPA (with 0.3% diethylamine, DEA) from 0–1 min; then linear increase to 75% CO_2 and 25% IPA (0.3% DEA) from 1–10 min; then linear decrease to 85% CO_2 and 15% IPA (0.3% DEA) from 10–12 min; keep at 85% CO_2 and 15% IPA (0.3% DEA) from 12–14 min. Retention times were 1.5 min biphenyl, 6.2 min for **4**, 8.6 min for (*R*)-**10**, 9.5 min for (*S*)-**10**. The compounds were quantified based on the relative substrate and product peak areas (absorbance at 280 nm) using a response factor of 2.9108.

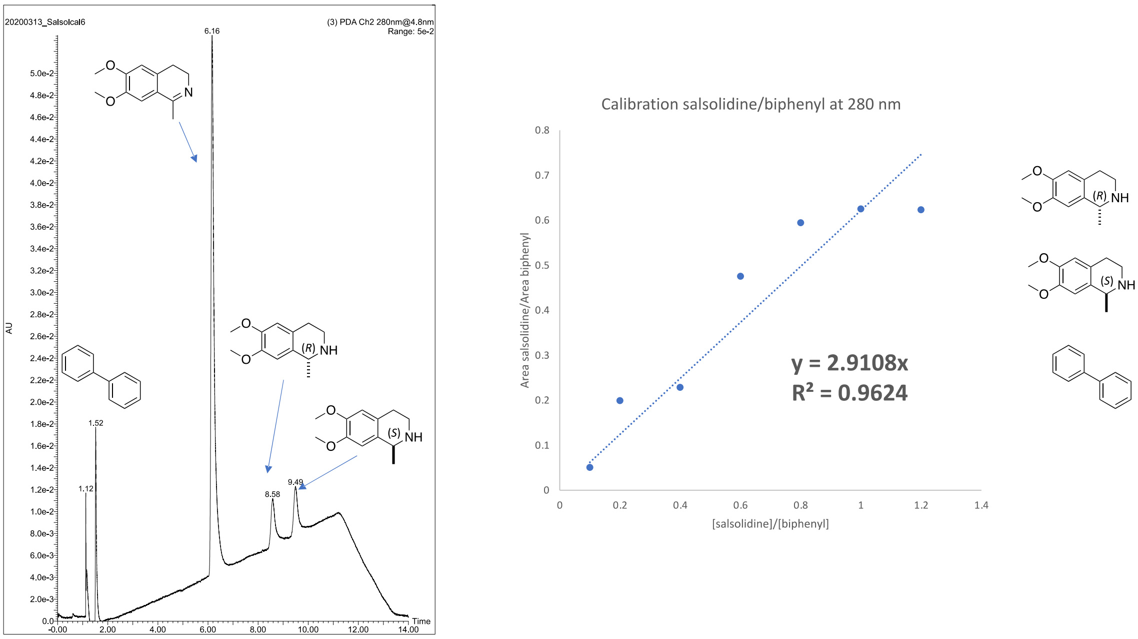

*Figure S10 Chromatogram and calibration curve for substrate **4**. Left: UPC2 chromatogram trace for the imine **4**, amine (*R*)- and (*S*)-**10** and the internal standard biphenyl in 10 mM concentrations. Right: The calibration curve for (*R*)- and (*S*)-**10** versus the internal standard biphenyl using the UPC2 method. The calibration was made in a range from 0.5 mM to 5 mM.*

Analysis of **5**, (*S*)-**11** and (*R*)-**11** was performed with a Chiralpak IA column (5 µm, 4.6 mm × 250 mm, Daicel) by UPC2. Biphenyl was used as internal standard. The program was set up with 80% CO_2 and 20% IPA (0.1% DEA) at a constant flow rate (2.5 mL/min). The retention times were 1.7 min for biphenyl, 2.4 min for **5**, 5.0 min for (*R*)-**11**, 8.1 min for (*S*)-**11**. The compounds were quantified based on the relative substrate and product peak peak areas and the internal standard area (absorbance at 210 nm) using a response factor of 1.2368.

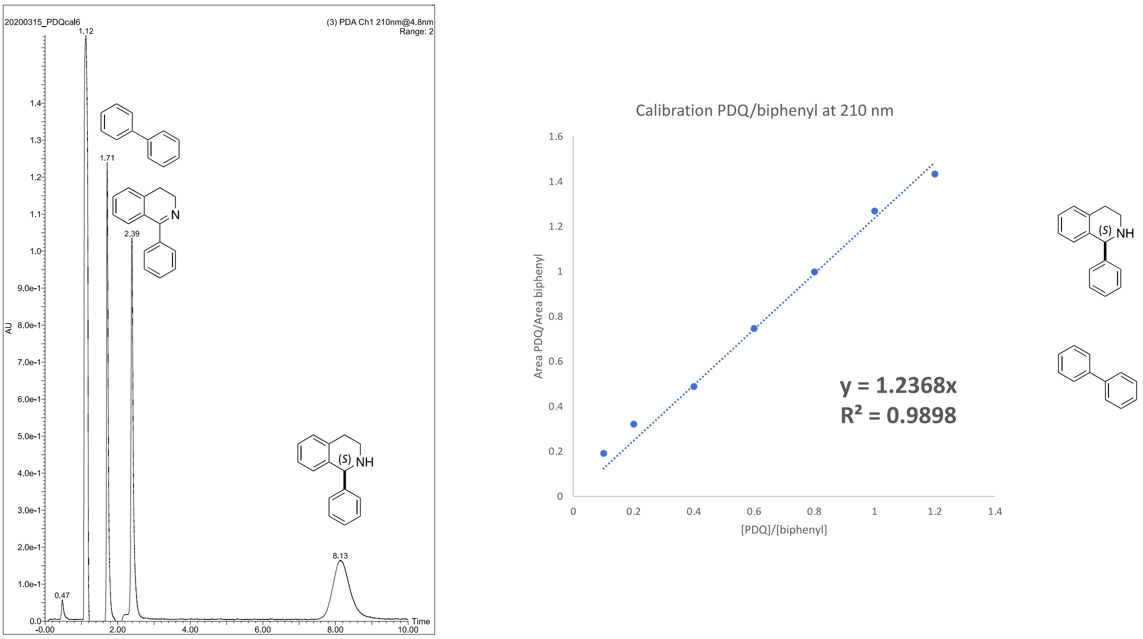

*Figure S11 Chromatogram and calibration curve for substrate **5**. Left: UPC2 chromatography trace for the imine **5**, amine (S)-**11** and the internal standard biphenyl in 10 mM concentrations. Right: The calibration curve for (S)-**11** versus internal standard biphenyl using the UPC2 method. The calibration was made in a range from 0.5 mM to 5 mM.*

Analysis of **6**, (*S*)-**12** and (*R*)-**12** was performed with a Chiralpak ID column (5 µm, 4.6 mm × 250 mm, Daicel) by UPC2. 2-methylindoline was used as an internal standard. The program was set up as follows: 80% CO_2 and 20% IPA (with 0.3% diethylamine, DEA) at a constant flow rate (2.5 mL/min). Retention times: 1.77 min for 2-methylindoline, 7.9 min for (*1*)-**12** and 8.9 for (*2*)-**12**. The compounds were quantified based on the relative peak areas and the internal standard area (absorance at 286 nm) using a response factor of 0.7281.

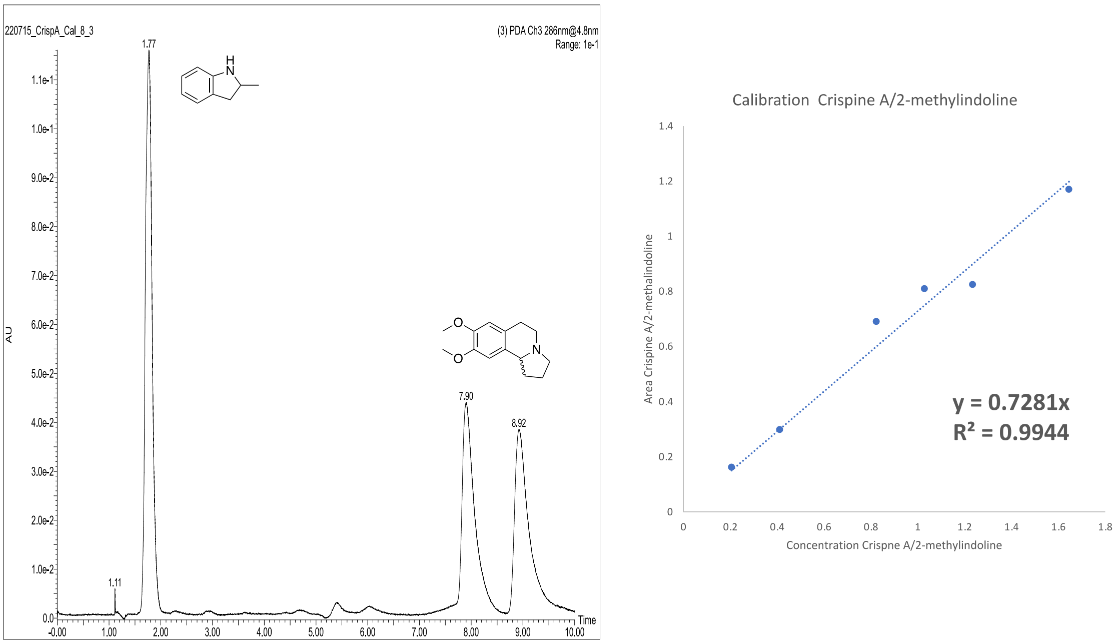

*Figure S12 Chromatogram and calibration curve for substrate **12**. Left: UPC2 chromatography trace for the amines **12** and the internal standard 2-methylindoline in 10 mM concentrations. Right: The calibration curve for **12** versus the internal standard 2-methylindoline using the UPC2 method. The calibration was made in a range from 0.5 mM to 5 mM.*

Analysis of **7**, (*S*)-**13** and (*R*)-**13** was performed with a Chiralpak ID column (5 µm, 4.6 mm × 250 mm, Daicel) by UPC2. 2-methylindoline was used as an internal standard. The program was set up as follows: 85% CO_2 and 15% IPA (with 0.3% diethylamine, DEA) from 0–1.5 min; then linear increase to 63% CO_2 and 37% IPA (0.3% DEA) from 1.5–2.3 min; this ratio was kept to 6 min; then the ratio was linear decrease to 85% CO_2 and 15% IPA (0.3% DEA) from 6–6.2 min; keep at 85% CO_2 and 15% IPA (0.3% DEA) from 6.2–6.5 min. Retention times: 2.0 min for 2-methylindoline, 4.6 min for **7**, 5.4 min for (*R*)-**13** and 5.8 for (*S*)-**13**. The compounds were quantified based on the relative substrate and product peak peak areas and the internal standard area (absorbance at 293 nm) using a response factor of 6.1247.

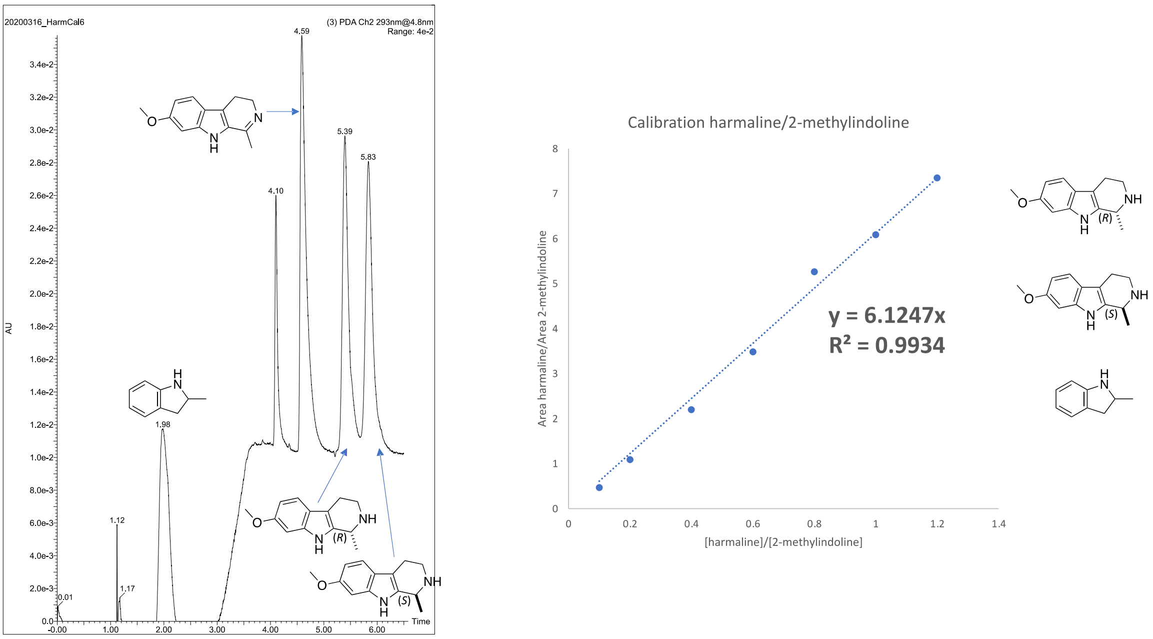

*Figure S13 Chromatogram and calibration curve for substrate **7**. Left: UPC2 chromatography trace for the imine **7**, amine (R)-**13** and (S)-**13** and the internal standard 2-methylindoline in 10 mM concentrations. Right: The calibration curve for (R)-**13** and (S)-**13** versus the internal standard 2-methylindoline using the UPC2 method. The calibration was made in a range from 0.5 mM to 5 mM.*

References

[1] C. Letondor, A. Pordea, N. Humbert, A. Ivanova, S. Mazurek, M. Novic, T. R. Ward, *J. Am. Chem. Soc.* **2006**, *128*, 8320–8328.

[2] H. Mallin, M. Hestericová, R. Reuter, T. R. Ward, *Nat. Protoc.* **2016**, *11*, 835–852.

[3] V. Chu, P. S. Stayton, S. Freitag, I. Le Trong, R. E. Stenkamp, *Protein Sci.* **1998**, *7*, 848–859.

[4] F. Christoffel, N. V. Igareta, M. M. Pellizzoni, L. Tiessler-Sala, B. Lozhkin, D. C. Spiess, A. Lledós, J.-D. D. Maréchal, R. L. Peterson, T. R. Ward, *Nat. Catal.* **2021**, *4*, 643–653.

[5] L. Potterton, J. Agirre, C. Ballard, K. Cowtan, E. Dodson, P. R. Evans, H. T. Jenkins, R. Keegan, E. Krissinel, K. Stevenson, A. Lebedev, S. J. McNicholas, R. A. Nicholls, M. Noble, N. S. Pannu, C. Roth, G. Sheldrick, P. Skubak, J. Turkenburg, V. Uski, F. Von Delft, D. Waterman, K. Wilson, M. Winn, M. Wojdyr, *Acta Crystallogr. Sect. D Struct. Biol.* **2018**, *74*, 68–84.

[6] https://server.poissonboltzmann.org/pdb2pqr
